# Perceptions of Pharmacology Teaching Approaches in Pre‐Doctoral Dental Education

**DOI:** 10.1002/jdd.70107

**Published:** 2025-11-24

**Authors:** Scott D. Papineau, Joshua D. Kellenberger, Timothy J. Treat

**Affiliations:** ^1^ Department of Biomedical and Applied Sciences Indiana University School of Dentistry Indianapolis Indiana USA; ^2^ Indiana University School of Dentistry Indianapolis Indiana USA; ^3^ Department of Comprehensive Care and Allied Professions Indiana University School of Dentistry Indianapolis Indiana USA

## 1 Problem

In health professions education, pharmacology is traditionally taught in lecture‐based didactic courses, which may not be integrated with clinical experiences [1]. Students struggle with a traditional didactic approach, as it often creates a disconnect between memorizing information and truly understanding its clinical relevance and application in patient care [2]. On course evaluations, many students report feeling overwhelmed by the volume of drug names and mechanisms they must memorize, often without a clear connection to real‐world patient care. As a result, their confidence in applying pharmacology knowledge in clinical decision‐making remains low.

Evidence of this is seen daily in the clinics of Indiana University School of Dentistry (IUSD), where students struggle to translate the drug facts learned in their pharmacology courses into practical decision‐making for the real patients sitting in their dental chairs. Our faculty members have noted that students frequently struggle to determine appropriate drug choices and fail to identify potential interactions, pertinent medication side effects, and contraindications in real‐time patient scenarios. These challenges highlight the urgent need for a more clinically integrated pharmacology curriculum.

## 2 Solution

In 2024, a new direction and lecture format were introduced for the pharmacology curriculum at IUSD. The new approach emphasized clinical relevance and incorporated case‐based scenarios with direct dental applications into every lecture with the intent of making the lectures more meaningful and engaging for dental students. The IUSD DDS Class of 2025 completed two pharmacology courses in back‐to‐back semesters during their third year. The first course, D711‐Pharmacotherapeutics I, utilized the traditional method, which was fact‐centered and relied heavily on memorization. The second course, D712‐Pharmacotherapeutics II, emphasized clinical relevance and incorporated case‐based scenarios with direct dental applications. A questionnaire was developed to understand their perceptions of these approaches. A 10‐question, 5‐point Likert‐scale questionnaire (Table 1) was distributed to 112 fourth‐year pre‐doctoral dental students who had completed both pharmacotherapeutics courses during their third year. The questionnaire was not IRB‐approved and was originally intended to collect student feedback to guide positive curriculum changes within the D711‐ and D712‐Pharmacotherapeutics courses.

**TABLE 1:** Perceptions of pharmacology teaching approaches in pre‐doctoral education.

	Student response				
Survey questions	Strongly agree	Agree	Neutral	Disagree	Strongly disagree
**1**. When comparing the two different teaching methods used to teach pharmacology in D711 and D712, the newer clinically relevant approach to teaching pharmacology helped me better understand the connection between pharmacology and dentistry.	84% (77)	15% (14)	1% (1)	0	0
**2**. When comparing the two different teaching methods used to teach pharmacology in D711 and D712, the newer approach helped me feel more confident in making pharmacology‐related decisions for my dental patients.	78% (72)	20% (18)	2% (2)	0	0
**3**. When comparing the two different teaching methods used to teach pharmacology in D711 and D712, I retained dentally important pharmacology concepts better with the clinically relevant teaching approach compared to the fact‐centered approach.	79% (73)	20% (18)	1% (1)	0	0
**4**. The inclusion of dental case scenarios in the new D712‐Pharmacotherapeutics course made the pharmacology concepts more applicable to real‐world dentistry when compared to the material presented in D711‐Pharmacotherapeutics.	89% (82)	11% (10)	0	0	0
**5**. The addition of images and dental‐specific examples in the new D712‐Pharmacotherapeutics course made the pharmacology material more engaging and easier to understand when compared to the material presented in D711‐Pharmacotherapeutics.	80% (74)	19% (17)	1% (1)	0	0
**6**. The newer approach to pharmacology education presented in D712‐Pharmacotherapeutics helped me feel more prepared to apply pharmacology concepts in patient care when compared to the approach used in D711‐Pharmacotherapeutics.	76% (70)	20% (18)	4% (4)	0	0
**7**. Compared to the previous fact‐centered, memorization‐based approach utilized in D711‐Pharmacotherapeutics, the newer approach utilized in D712‐Pharmacotherapeutics helped me better appreciate the role of pharmacology in dentistry.	75% (69)	22% (20)	3% (3)	0	0
**8**. I believe future dental students would benefit from a clinically relevant, case‐based approach to pharmacology education when compared to the more traditional fact‐based, memorization approach previously utilized.	89% (82)	11% (10)	0	0	0
**9**. After experiencing both teaching styles (D711 and D712), I now see how the educational approach to teaching pharmacology plays a critical role in dental student comprehension and application of pharmacology concepts.	80% (74)	19% (17)	1% (1)	0	0
**10**. If given the choice, I would prefer the dentally focused, clinically relevant approach to pharmacology utilized in D712‐Pharmacotherapeutics over the fact‐centered, memorization‐based approach utilized in D711‐Pharmacotherapeutics.	86% (79)	14% (13)	0	0	0

## 3 Results

The questionnaire had a response rate of 82% (92 responses). All respondents (100%) preferred the newer teaching method over the traditional approach, with 86% selecting “strongly agree.” After experiencing both teaching approaches, 100% of respondents agreed that their ability to apply key pharmacology concepts improved. In comparison, 99% felt their retention of critical pharmacology concepts was enhanced with the newer, clinically relevant teaching modality.

In addition, 99% of respondents reported that the case‐based approach made pharmacology material more engaging and easier to understand. When asked if the clinically focused method boosted their confidence in patient care, 98% responded affirmatively. All respondents (100%) believed that future dental students would benefit more from the case‐based teaching approach.

## 4 Conclusion

This study suggests that a case‐based, clinically relevant teaching approach improves students’ confidence in both understanding pharmacology concepts and applying them in clinical decision‐making. These findings support continued implementation of this instructional method within the IUSD pharmacology curriculum. Future studies should include both subjective and objective comparisons between cohorts trained exclusively with this approach and those educated through traditional didactic methods to evaluate long‐term retention, clinical performance, and overall educational effectiveness.

## References

[1] F. Engels , “Pharmacology Education: Reflections and Challenges,” European Journal of Pharmacology 833 (2018): 392–395, 10.1016/j.ejphar.2018.06.032. 29935169

[2] C. J. Miller , J. Mcnear , and M. J. Metz , “A Comparison of Traditional and Engaging Lecture Methods in a Large, Professional‐Level Course,” Advances in Physiology Education 37, no. 4 (2013): 347–355, 10.1152/advan.00050.2013. 24292912

